# Effect of Smoking Behavior before and during Pregnancy on Selected Birth Outcomes among Singleton Full-Term Pregnancy: A Murmansk County Birth Registry Study

**DOI:** 10.3390/ijerph14080867

**Published:** 2017-08-02

**Authors:** Olga A. Kharkova, Andrej M. Grjibovski, Alexandra Krettek, Evert Nieboer, Jon Ø. Odland

**Affiliations:** 1 Department of Community Medicine, Faculty of Health Sciences, UiT The Arctic University of Norway, 9037 Tromsø, Norway; alexandra.krettek@uit.no (A.K.); jon.oyvind.odland@uit.no (J.Ø.O.); 2 International School of Public Health, Northern State Medical University, Arkhangelsk 163000, Russia; andrej.grjibovski@gmail.com; 3 Department of Public Health, North-Eastern Federal University, Yakutsk 677000, Russia; 4 Department of Preventive Medicine, International Kazakh-Turkish University, Turkestan 161200, Kazakhstan; 5 Department of Biomedicine and Public Health, School of Health and Education, University of Skövde, 54128 Skövde, Sweden; 6 Department of Internal Medicine and Clinical Nutrition, Institute of Medicine, Sahlgrenska Academy, University of Gothenburg, 41390 Gothenburg, Sweden; 7 Department of Biochemistry and Biomedical Sciences, McMaster University, Hamilton, ON 2303, Canada; nieboere@mcmaster.ca; 8 School of Health Systems and Public Health, Faculty of Health Sciences, University of Pretoria, Pretoria L8S4L8, South Africa

**Keywords:** smoking, cigarettes, smoking cessation, low birth weight, low birth length, low head circumference, low ponderal index, low Apgar score at 5 min, Murmansk County Birth Registry, Russia

## Abstract

The aim of our study was to assess associations between smoking behavior before and during pregnancy and selected adverse birth outcomes. This study is based on the Murmansk County Birth Registry (MCBR). Our study includes women who delivered a singleton pregnancy after 37 weeks of gestation (N = 44,486). Smoking information was self-reported and assessed at the first antenatal visit during pregnancy. We adjusted for potential confounders using logistic regression. The highest proportion of infants with low values of birth weight, birth length, head circumference, ponderal index and of the Apgar score at 5 min was observed for women who smoked both before and during pregnancy. We observed a dose-response relationship between the number of cigarettes smoked per day during pregnancy and the odds of the aforementioned adverse birth outcomes; neither were there significant differences in their occurrences among non-smokers and those who smoked before but not during pregnancy. Moreover, smoking reduction during pregnancy relative to its pre-gestation level did not influence the odds of the adverse birth outcomes. Our findings emphasize a continued need for action against tobacco smoking during pregnancy.

## 1. Introduction

Tobacco smoking is a public health problem. Even though this habit is less common among women than men in Russia, it appears to be on the increase among women aged ≥15 years [1]. Consequently, this trend will lead to an increased prevalence of smoking during pregnancy. At the end of the 20th century, the maternal smoking rate in Russia was 16.3% [2], while in 2006–2011 it was 18.9% [3].

Smoking during pregnancy is known to impair placental development by reducing blood flow [4]. It can produce a hypoxic environment and lead to a reduced provision of oxygen and micronutrients. Its adverse effects on pregnancy and birth outcomes include placenta previa [5] and placental abruption [6], as well as ectopic pregnancy [7] and miscarriage [8]. The incidence of preterm deliveries and the incidence of very-early preterm deliveries are also reported to be higher in women who smoke during pregnancy [9].

Birth weight, length and head circumference at birth are major indices of fetal growth that maternal smoking appears to suppress [10]. Compared to the number of studies on low birth weight [11,12,13,14,15], the influences of quitting smoking or smoking reduction during pregnancy on birth length [10,16] and head circumference [17,18] are not as well documented.

The ponderal index is a measure of birth weight in relation to crown-heel length [19]. It is used as a proxy for body composition to assess growth abnormalities of infants. Asymmetric infants are either thinner and have less birth weight per centimeter of length (i.e., low ponderal index), or are shorter and have high birth weight per centimeter of length (high ponderal index) than symmetrical newborns. However, there is no consistent evidence to determine if smoking or giving it up during pregnancy influences this variable. Some studies demonstrate no statistical association [19,20], whereas others indicate a reduction [21,22].

The Apgar score is widely used as a standardized index of the newborn health status in the immediate neonatal period [23]. A low Apgar score (i.e., <7) is strongly associated with a risk of neonatal and infant death [24]. Walfisch et al. [25] report that babies of smoking mothers had lower Apgar scores at 5 min compared to those of non-smokers, although smoking during pregnancy was not an independent predictor of the Apgar score. Moreover, it is unclear whether giving up smoking during pregnancy affects the Apgar score.

Based on the evidence reviewed, enough uncertainty remains to warrant further examination of the impact of smoking on birth weight, length, head circumference, the ponderal index and Apgar score. We hereby do so by comparing their respective odds ratio for smokers, those who reduced the number of cigarettes smoked during pregnancy, and those who smoked before pregnancy but stopped doing so during the first trimester.

## 2. Materials and Methods

### 2.1. Study Setting, Design and Sample Size

The Murmansk County Birth Registry (MCBR) contains detailed information on more than 99% of all births in Murmansk County (Northwest Russia) during the period 2006–2011. The MCBR was a cooperative effort between the University of Tromsø (Norway), Murmansk County Health Department and all delivery departments in Murmansk County. Detailed information about its design and implementation has been provided previously [26].

**Figure 1:**
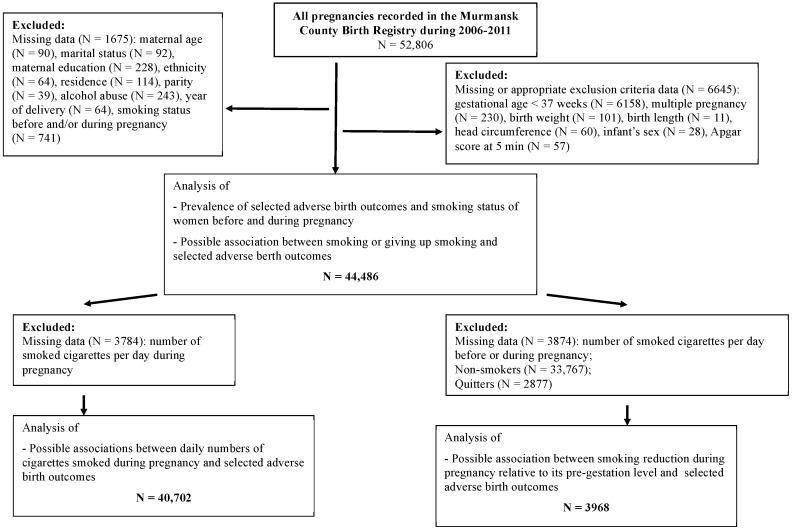
Study population selection procedure.

A total of 52,806 pregnancies were registered in the MCBR from 1 January 2006 to 31 December 2011. For the purpose of this study, we excluded women if they had delivered before 37 completed weeks of gestation or had a multiple pregnancy. Our study focused on three main tobacco-smoking issues related to pregnancy: (i) smoking status; (ii) number of cigarettes smoked daily; and (iii) smoking reduction compared to its pre-gestation frequency. Sampling details and missing data are summarized in Figure 1. The exclusion criteria indicated in this figure are as adopted previously [3].

### 2.2. Data Collection

Based on medical records and personal interviews with expecting mothers, the MCBR contains information on maternal characteristics including age, ethnicity, residence, educational level, marital status, parity, alcohol abuse as diagnosed by a doctor, self-reported smoking (number of cigarettes per day before and during pregnancy), and maternal weight and height measured at the first antenatal visit. Information in the MCBR on gestational diabetes, excessive weight gain during pregnancy, gestational age and year of delivery was derived from individual obstetric journals. Based on newborn delivery records, the MCBR also contains data about birth weight, length, head circumference and Apgar score at 5 min.

### 2.3. Dependent Variable

Low birth weight, length and head circumference were defined according to the World Health Organization as mean values minus 2 standard deviations (M-2SD) for girls and boys separately [27]. Respectively for girls and boys, low birth weight was <2400 g and <2500 g; low birth length <45.4 cm and <46.1 cm; and low birth head circumference <31.5 cm and <31.9 cm.

We used the ponderal index in newborns to assess asymmetrical intrauterine growth retardation (IGR). This was defined as 100 × birth weight (g)/length^3^ (cm), and a low score below the 10th centile (<2.14) was taken as an estimate of disproportionate IGR. The Apgar score at 5 min is a combined score of five readily identifiable neonatal characteristics that includes skin color, heart rate, respiratory effort, muscle tone, and reflexes [23]. Scores of six or lower are considered low.

### 2.4. Independent Variables

Smoking status during pregnancy included the variables: ‘Smoking before pregnancy’ and ‘Smoking during pregnancy’. Women who smoked before and during pregnancy were designated as *smokers*, those who did so before but not during pregnancy were defined as *quitters*, and as *non-smokers* when they neither smoked before or during pregnancy. Number of cigarettes smoked per day during pregnancy was taken as a categorical variable, specifically as 0, 1–5, 6–10, and ≥11. Smoking reduction during pregnancy relative to its pre-gestation level was dichotomized as “Yes” and “No”. The latter included women who increased the number of cigarettes smoked per day during pregnancy, as well as those who did not change their smoking pattern. Smoking status was assessed during the first antenatal visit.

Adjustments were made for maternal age, place of residence, ethnicity, maternal education, marital status, year of delivery, parity, body mass index at the first antenatal visit, gestational diabetes, excessive weight gain in pregnancy, gestational age and alcohol abuse.

### 2.5. Data Analysis

Categorical variables are presented as numbers or percentages and Pearson’s Chi-square tests were used to assess statistical significance of differences. By logistic regression, we examined the associations between several adverse birth outcomes and smoking status during pregnancy, the number of cigarettes smoked per day during pregnancy, as well as reduction in smoking while pregnant. Crude and adjusted odds ratios (OR) were calculated with 95% confidence intervals (CI). We tested for trends by entering ordinal variables as continuous in the regression analyses. All statistical analyses were conducted using SPSS version 23 (SPSS Inc., Chicago, IL, USA).

### 2.6. Ethical Considerations

This study was approved by the Ethical Committees of the Northern State Medical University, Arkhangelsk (Russia) (identification code: No. 08/12-14 from 10.12.2014) and the Norwegian Regional Committee for Medical and Health Research Ethics (REC-North), Tromsø (Norway) (identification code: No. 2014/1660).

## 3. Results

### 3.1. Prevalence of Selected Adverse Birth Outcomes and Smoking Behaviour of Women before and during Pregnancy

The prevalence of neonatal indices with low values included: birth weight (1.1%), birth length (0.6%), head circumference (2.4%), ponderal index (11.0%), and Apgar score at 5 min (1.0%). These adverse birth outcomes were more prevalent in women who smoked during pregnancy (Table 1) and their proportions increased with the number of cigarettes smoked per day during pregnancy (for trend *p* < 0.001), with ponderal index the exception. For the latter, the highest proportion of newborns with a low value was most common among women who smoked 1–5 cigarettes per day during pregnancy, while the *lowest* proportion occurred among those who smoked ≥11 cigarettes daily.

### 3.2. Association between Daily Numbers of Smoked Cigarettes during Pregnancy and Selected Adverse Birth Outcomes among Women with Singleton Full-Term Pregnancies

Associations between daily numbers of smoked cigarettes during pregnancy and selected adverse birth outcomes are presented in Table 2.

A dose-response relationship is evident between the number of cigarettes smoked per day during pregnancy and the odds of low birth weight, low birth length, low head circumference, low ponderal index and low Apgar score at 5 min. Adjustment for potential confounders did not change these associations. Respectively, mothers who smoked ≥11 cigarettes per day while pregnant were 2.1, 5.4, 5.2 and 2.1 times more likely to deliver an infant with low values of birth weight, birth length, head circumference and Apgar score at 5 min compared to non-smokers (see adjusted OR_≥11 cigarettes_ in Table 2). Women who smoked 1–5 cigarettes per day during pregnancy had a higher odds of having a low ponderal-index infant compared to non-smokers (before and after adjustment for confounders; adjusted OR_1–5 cigarettes_ of 1.57 with 95% CI: 1.38–1.80), while those who smoked ≥11 cigarettes per day during pregnancy were almost two-fold less likely to have such infant (before and after adjustment; adjusted OR_≥11 cigarettes_ of 0.56 with 95% CI: 0.40–0.80).

### 3.3. Association of Selected Adverse birth Outcomes and Smoking, Giving-Up Smoking, or Smoking Reduction

Compared to non-smokers in the crude analysis summarized in Table 3, low birth weight and low birth length were almost three times more likely among smokers (both before and during pregnancy).

Similarly, their babies had higher odds of having a low head circumference, low ponderal index or low Apgar score at 5 min. After adjustment for confounders, the statistical significance for the Apgar score was lost. In addition and relative to non-smokers (see Table 3), interruption of smoking during pregnancy had no significant impact on the adverse birth outcomes considered (prior and subsequent to adjustments for potential confounders). Moreover, smoking reduction during pregnancy did not alter the odds of the selected adverse birth outcomes (Table 4).

## 4. Discussion

### 4.1. Main Findings

The highest proportion of infants with low values of birth weight, birth length, head circumference, low ponderal index, and Apgar score at 5 min was observed among women who smoked both before and during pregnancy. A dose-response relationship was evident between numbers of cigarettes smoked daily during pregnancy and selected adverse birth outcomes. Cessation of smoking during pregnancy reduces the risks to the levels for non-smoking women. By contrast, smoking reduction during pregnancy relative to its pre-gestation frequency did not reduce the risks considered.

### 4.2. Data Interpretation and Comparisons with Previous Studies

#### 4.2.1. Smoking before and during Pregnancy

A baby’s low weight at birth is either the result of preterm birth (before 37 weeks of gestation) or due to restricted fetal growth [28]. Consequently, we limited our study to births after the 37th week. Perhaps this explains the unexpectedly low prevalence of infants having low birth weight in our study in comparison with other studies that include preterm births and multiple pregnancies [11,13,15,29]. Our observation that risk of low birth weight was associated with maternal smoking agrees with earlier studies [10,11,15,16,29,30].

Kato et al. [31] indicate that birth length is an important predictor of subsequent health. In our study, less than 1.0% of term infants had low birth length that was associated with smoking during pregnancy. Nevertheless, low birth length was almost three times higher among smokers compared to non-smokers. Inoue et al. [10] observed the same outcome. Similarly, other studies have reported that children from mothers who continued smoking during pregnancy were shorter until the age of 4 years [16,19,32].

Several reports identify reduced head circumference and biparietal diameter as parameters of total growth restriction in fetuses of smoking mothers [10,18,32,33]. We found an association between low head circumference at birth and maternal smoking. It has been suggested that this association is not only due to premature birth and smoking during pregnancy, but also by a negative effect of maternal smoking on intrauterine head growth [17]. Fattal-Valevski et al. [34] indicate that head size is an index of abnormal brain condition or neurodevelopmental delay in cognitive functions, and therefore reflects a child’s long-term cognitive outcome [34].

Our adjusted odds for asymmetrical infants was 15% higher among women who smoked both before and during pregnancy compared to non-smokers. Previous studies with the ponderal index as a continuous variable have demonstrated decreases in its mean with maternal smoking [22,35], although Ingvarsson et al. [19] report no such relationship.

The absence of an association between maternal smoking and the odds of having infants with low Apgar score at 5 min might have been influenced by the fact that we focused on term births only. Walfisch et al. [25] also observed a non-significant association. Furthermore, a study of tobacco biomarkers in meconium did not observe an association between low Apgar score at 5 min and maternal smoking [36].

#### 4.2.2. Daily Number of Cigarettes Smoked during Pregnancy

The dose-response relationship we demonstrate between daily number of cigarettes smoked during pregnancy and adverse birth outcomes is supported by earlier reports. Our finding is comparable to that indicated by Ko et al. [13], namely OR_adjusted_ = 2.48 with 95% CI = 1.76–3.49). Ward et al. [15] have investigated the dependence of birth weight on cigarette smoking and observed a linear trend for reduced birth weight with increasing level of exposure involving either environmental tobacco smoke exposure (only partner smoked during the pregnancy) and for maternal smoking. Comparable findings have been reported by Durmus et al. [16] and Wang et al. [12]. Even though the study by Lindley et al. [22] comprised singleton births with gestational ages of more than 24 weeks, they also demonstrated that moderate maternal smoking was associated with a decrease in mean crown-heel length of 0.63 cm, while heavy smoking was related with a decline of 0.84 cm.

The number of studies examining dose-response relationships between daily cigarettes smoked during pregnancy and other anthropometric parameters of the newborn is limited. Jaddoe et al. [18] investigated associations of maternal smoking during pregnancy with longitudinally measured fetal growth characteristics, in particular head circumference for mid- and late gestations. The largest impact was observed in late gestation for the highest smoking category, namely ≥9 cigarettes per day [18]. Also in a large Swedish birth cohort of 1,362,169 infants, significant dose-response effects were observed for the effect of maternal smoking on head circumference <32 cm and less than the mean-2SD of its expected value [17].

Lindley et al. [22] also demonstrated that compared to non-smokers, heavy maternal smoking was associated with an increase in the ponderal index of 0.04. Thus infants of heavy smokers are more symmetrical in their growth retardation than those of light smokers. It is considered that the neonatal morbidity rate for symmetrical IGR is higher than that for asymmetrical IGR, and that term symmetric infants with IGR tend to have a lower mean birth weight implying a higher incidence of small placentas than for term infants with asymmetrical IGR [37]. It may be concluded that heavy smoking during pregnancy relative to light smoking leads to a reduction in a newborn’s health.

We did not find an association between low Apgar scores at 5 min and maternal smoking. However, a dose-response relationship between these variables was evident. Most of the studies estimating dose-responses were done more than 20 years ago and showed differential results. For example one study suggested a negative influence of maternal smoking on Apgar score at 5 min [38], while others showed no effect [39,40].

#### 4.2.3. Giving up Smoking in the First-Trimester

We observed that women who stop smoking after pregnancy recognition are at no greater risk of having a term baby with all selected adverse birth outcomes compared to non-smokers. Nijiati et al. [41] also showed that mean birth weight is not significantly different when comparing participants who stop smoking during pregnancy to non-smoking participants, and therefore conclude that smoking cessation in pregnancy is beneficial. By contrast, others have reported that maternal smoking in the first trimester is not associated with growth differences in head circumferences, lengths, and weight when compared to non-smokers [16,42].

#### 4.2.4. Smoking Reduction during Pregnancy Compared to Pre-Gestation Level

The lack of an effect of reduced smoking observed may have been limited by a number of factors, including the accuracy/completeness of our data on smoking, heterogeneity of mitigating factors and the relatively low number of cigarettes smoked daily by Russian women. However, in some studies a statistical association between reduction number of cigarettes smoked per day during pregnancy and birth weight has not been observed [14,43,44].

### 4.3. Limitations and Strengths

Our information on smoking behavior before and during pregnancy was based on self-reporting and assessed only in the first antenatal visit in the first trimester. Consequently, underreporting of maternal smoking across different smoking categories may have occurred and led to misclassification. Tobacco smoking in Russia during the study period was restricted for children, and its use during pregnancy was not discouraged. Meta-analyses of studies comparing self-reported smoking with biochemical assessments have concluded that self-reports of smoking are accurate in most studies and are sufficiently sensitive and specific [45].

In our study, information about smoking reduction during pregnancy compared to before gestation was missing for 49.4% of all smokers. This nonresponse may have led to bias. Nevertheless, our observation of no association between a reduction in the daily number of cigarettes smoked during pregnancy and risk of infants with low birth weight is supported by previous studies [14,43,44]. Since smoking reduction was based on a dichotomous variable (yes/no), an attenuation effect may have occurred.

The major strength of our study is that it is based on birth registry data, and thereby included both socio-demographic and clinical characteristics of the women. Even though the registry data were collected in clinics, it corresponds to 98.8% of the official number of births recorded by the Murmansk County Health Department [26]. This allowed the generalization of the results to the population level. Since the data on the quantity of cigarettes smoked before and during pregnancy were collected as part of the MCBR data collection, we were able to evaluate dose-response relationships of maternal smoking and the effect of smoking reduction during pregnancy compared to before gestation on the prevalence of selected adverse birth outcomes.

## 5. Conclusions

In conclusion, women who stopped smoking during the first trimester were at no higher risk of having a baby with the selected adverse birth outcomes as compared to non-smokers. Of special interest is that a smoking reduction during pregnancy was not associated with a reduction in the adverse birth outcomes examined, although the limited statistical power for this aspect of our work cannot be precluded. Our study illustrates that smoking before and during pregnancy leads to infants with reduced birth weight, birth length, head circumference, and ponderal index. Moreover, dose-response relationships were observed between maternal smoking and these adverse birth outcomes. Our study findings reemphasize the need for continued action against tobacco smoking during pregnancy.

## Figures and Tables

**Table 1:** Smoking behavior of women with spontaneous singleton births and selected adverse birth outcomes in Murmansk County, Northwest Russia.

Smoking Behavior of Pregnant Women	Low Birth Weight			Low Birth Length			Low Head Circumference			Low Ponderal Index			Low Apgar Score at 5 min		
	N	%	*p*	N	%	*p*	N	%	*p*	N	%	*p*	N	%	*p*
*Smoking status during pregnancy (N = 44,486)*			<0.001			<0.001			<0.001			0.002			0.065
Non-smoker	290	0.9		142	0.4		673	2.0		3611	10.7		305	0.9	
Quitter	24	0.8		13	0.5		66	2.3		323	11.2		26	0.9	
Smoker	194	2.5		98	1.2		338	4.3		947	12.1		93	1.2	
*Number of smoked cigarettes per day during pregnancy (N = 40,702)*			<0.001			<0.001			<0.001			<0.001			<0.001
0	314	0.9		155	0.4		739	2.0		3933	10.7		331	0.9	
1–5	40	2.2		20	1.1		69	3.8		298	16.4		23	1.3	
6–10	38	2.2		26	1.5		84	5.0		186	11.0		29	1.7	
≥11	17	3.1		19	3.4		65	11.7		35	6.3		11	2.0	
*Smoking reduction during pregnancy relative to its pre-gestation level (N = 3968)*			0.176			0.208			0.208			0.156			0.572
No	61	2.5		43	1.8		140	5.7		295	12.1		41	1.7	
Yes	28	1.8		19	1.2		73	4.8		207	13.6		22	1.4	

Calculated using chi-squared test.

**Table 2:** Association between daily numbers of smoked cigarettes during pregnancy and selected adverse birth outcomes among women with singleton full-term pregnancy in Murmansk County, Northwest Russia (N = 40,702).

Adverse Birth Outcome	Crude OR (95% CI)				Adjusted OR (95% CI) ^1^			
	Daily Numbers of Smoked Cigarettes during Pregnancy				Daily Numbers of Smoked Cigarettes during Pregnancy			
	0	1–5	5–10	≥11	0	1–5	5–10	≥11
Low birth weight	1.00	2.60 (1.87–3.63)	2.66 (1.89–3.73)	3.66 (2.23–6.0)	1.00	2.02 (1.43–2.86)	1.80 (1.25–2.58)	2.06 (1.19–3.58)
Low birth length	1.00	2.62 (1.64–4.18)	3.67 (2.42–5.58)	8.36 (5.15–13.6)	1.00	2.25 (1.38–3.68)	2.75 (1.76–4.30)	5.36 (3.08–9.32)
Low head circumference	1.00	1.92 (1.49–2.46)	2.54 (2.01–3.20)	6.46 (4.93–8.45)	1.00	1.69 (1.31–2.19)	2.08 (1.63–2.65)	5.19 (3.89–6.92)
Low ponderal index	1.00	1.63 (1.43–1.85)	1.03 (0.88–1.20)	0.56 (0.40–0.79)	1.00	1.57 (1.38–1.80)	0.99 (0.84–1.16)	0.56 (0.40–0.80)
Low Apgar score at 5 min	1.00	1.41 (0.92–2.15)	1.91 (1.30–2.80)	2.22 (1.21–4.08)	1.00	1.35 (0.87–2.08)	1.83 (1.23–2.73)	2.06 (1.10–3.89)

^1^ OR was adjusted for the variables maternal age, residence, ethnicity, education, marital status, parity, alcohol abuse, year of delivery, body mass index, pregnancy diabetes, gestational age and excessive weight gain.

**Table 3:** Association between smoking status before and during pregnancy and selected adverse birth outcomes in Murmansk County, Northwest Russia (N = 44,486).

Adverse Birth Outcome	Crude OR (95% CI)			Adjusted OR (95% CI) ^1^		
	Smoking Status during Pregnancy			Smoking Status during Pregnancy		
	Non-Smoker	Quitter	Smoker	Non-Smoker	Quitter	Smoker
Low birth weight	1.00	0.97 (0.64–1.47)	2.92 (2.44–3.52)	1.00	0.89 (0.58–1.36)	2.10 (1.72–2.57)
Low birth length	1.00	1.07 (0.61–1.90)	3.00 (2.31–3.88)	1.00	1.09 (0.61–1.93)	2.36 (1.78–3.14)
Low head circumference	1.00	1.15 (0.89–1.49)	2.21 (1.94–2.53)	1.00	1.04 (0.80–1.34)	1.77 (1.53–2.04)
Low ponderal index	1.00	1.06 (0.94–1.19)	1.15 (1.06–1.24)	1.00	1.05 (0.93–1.19)	1.15 (1.06–1.24)
Low Apgar score at 5 min	1.00	1.00 (0.67–1.50)	1.32 (1.04–1.66)	1.00	0.94 (0.62–1.40)	1.24 (0.97–1.59)

^1^ OR adjusted for the variables maternal age, residence, ethnicity, education, marital status, parity, alcohol abuse, year of delivery, body mass index, pregnancy diabetes, gestational age, and excessive weight gain.

**Table 4:** Association between smoking reduction during pregnancy relative to its pre-gestation level and selected adverse birth outcomes in Murmansk County, Northwest Russia (N = 3968).

Adverse Birth Outcome	Crude OR (95% CI)		Adjusted OR (95% CI) ^1^	
	Smoking Reduction during Pregnancy		Smoking Reduction during Pregnancy	
	No	Yes	No	Yes
Low birth weight	1.00	0.73 (0.47–1.15)	1.00	0.87 (0.54–1.39)
Low birth length	1.00	0.71 (0.41–1.22)	1.00	0.83 (0.47–1.46)
Low head circumference	1.00	0.83 (0.62–1.11)	1.00	0.83 (0.62–1.12)
Low ponderal index	1.00	0.86 (0.51–1.45)	1.00	0.86 (0.50–1.46)
Low Apgar score at 5 min	1.00	1.15 (0.95–1.40)	1.00	1.10 (0.91–1.34)

^1^ OR was adjusted for the variables maternal age, residence, ethnicity, education, marital status, parity, alcohol abuse, year of delivery, body mass index, pregnancy diabetes, gestational age, and excessive weight gain.

## References

[1] Tobacco USE Data by Country. http://apps.who.int/gho/data/node.main.65.

[2] Grjibovski A., Bygren L.O., Svartbo B. (2002). Socio-demographic determinants of poor infant outcome in north-west Russia. Paediatr. Perinat Epidemiol..

[3] Kharkova O.A., Krettek A., Grjibovski A.M., Nieboer E., Odland J.O. (2016). Prevalence of smoking before and during pregnancy and changes in this habit during pregnancy in Northwest Russia: A Murmansk County Birth Registry study. Reprod. Health.

[4] Zdravkovic T., Genbacev O., McMaster M.T., Fisher S.J. (2005). The adverse effects of maternal smoking on the human placenta: A review. Placenta.

[5] Tikkanen M., Nuutila M., Hiilesmaa V., Paavonen J., Ylikorkala O. (2006). Prepregnancy risk factors for placental abruption. Acta Obstet. Gynecol. Scand..

[6] Oyelese Y., Smulian J.C. (2006). Placenta Previa, placenta accreta, and vasa Previa. Obstet. Gynecol..

[7] Saraiya M., Berg C.J., Kendrick J.S., Strauss L.T., Atrash H.K., Ahn Y.W. (1998). Cigarette smoking as a risk factor for ectopic pregnancy. Am. J. Obstet. Gynecol..

[8] Mishra G.D., Dobson A.J., Schofield M.J. (2000). Cigarette smoking, menstrual symptoms and miscarriage among young women. Aust. N. Z. J. Public Health.

[9] Jauniaux E., Burton G.J. (2007). Morphological and biological effects of maternal exposure to tobacco smoke on the feto-placental unit. Early Hum. Dev..

[10] Inoue S., Naruse H., Yorifuji T., Kato T., Murakoshi T., Doi H., Subramanian S.V. (2016). Impact of maternal and paternal smoking on birth outcomes. J. Public Health (Oxf.).

[11] Timur Taşhan S., Hotun Sahin N., Omaç Sönmez M. (2016). Maternal smoking and newborn sex, birth weight and breastfeeding: A population-based study. J. Matern.-Fetal Neonatal Med..

[12] Wang N., Tikellis G., Sun C., Pezic A., Wang L., Wells J.C., Cochrane J., Ponsonby A.L., Dwyer T. (2014). The effect of maternal prenatal smoking and alcohol consumption on the placenta-to-birth weight ratio. Placenta.

[13] Ko T.J., Tsai L.Y., Chu L.C., Yeh S.J., Leung C., Chen C.Y., Chou H.C., Tsao P.N., Chen P.C., Hsieh W.S. (2014). Parental smoking during pregnancy and its association with low birth weight, small for gestational age, and preterm birth offspring: A birth cohort study. Pediatr. Neonatol..

[14] Benjamin-Garner R., Stotts A. (2013). Impact of smoking exposure change on infant birth weight among a cohort of women in a prenatal smoking cessation study. Nicotine Tob. Res..

[15] Ward C., Lewis S., Coleman T. (2007). Prevalence of maternal smoking and environmental tobacco smoke exposure during pregnancy and impact on birth weight: Retrospective study using Millennium Cohort. BMC Public Health.

[16] Durmus B., Kruithof C.J., Gillman M.H., Willemsen S.P., Hofman A., Raat H., Eilers P.H., Steegers E.A., Jaddoe V.W. (2011). Parental smoking during pregnancy, early growth, and risk of obesity in preschool children: The Generation R Study. Am. J. Clin. Nutr..

[17] Kallen K. (2000). Maternal smoking during pregnancy and infant head circumference at birth. Early Hum. Dev..

[18] Jaddoe V.W., Verburg B.O., de Ridder M.A., Hofman A., Mackenbach J.P., Moll H.A., Steegers E.A., Witteman J.C. (2007). Maternal smoking and fetal growth characteristics in different periods of pregnancy: The generation R study. Am. J. Epidemiol..

[19] Ingvarsson R.F., Bjarnason A.O., Dagbjartsson A., Hardardottir H., Haraldsson A., Thorkelsson T. (2007). The effects of smoking in pregnancy on factors influencing fetal growth. Acta Paediatr..

[20] Grjibovski A., Bygren L.O., Svartbo B., Magnus P. (2004). Housing conditions, perceived stress, smoking, and alcohol: Determinants of fetal growth in Northwest Russia. Acta Obstet. Gynecol. Scand..

[21] Haste F.M., Anderson H.R., Brooke O.G., Bland J.M., Peacock J.L. (1991). The effects of smoking and drinking on the anthropometric measurements of neonates. Paediatr. Perinat. Epidemiol..

[22] Lindley A.A., Gray R.H., Herman A.A., Becker S. (2000). Maternal cigarette smoking during pregnancy and infant ponderal index at birth in the Swedish Medical Birth Register, 1991–1992. Am. J. Public Health.

[23] Apgar V. (1953). A proposal for a new method of evaluation of the newborn infant. Curr. Res. Anesth. Analg..

[24] Iliodromiti S., Mackay D.F., Smith G.C., Pell J.P., Nelson S.M. (2014). Apgar score and the risk of cause-specific infant mortality: A population-based cohort study. Lancet.

[25] Walfisch A., Nikolovski S., Talevska B., Hallak M. (2013). Fetal growth restriction and maternal smoking in the Macedonian Roma population: A causality dilemma. Arch. Gynecol. Obstet..

[26] Anda E.E., Nieboer E., Voitov A.V., Kovalenko A.A., Lapina Y.M., Voitova E.A., Kovalenko L.F., Odland J.Ø. (2008). Implementation, quality control and selected pregnancy outcomes of the Murmansk County Birth Registry in Russia. Int. J. Circumpolar Health.

[27] WHO. http://www.who.int/childgrowth/standards/ru/.

[28] United Nations Children’s Fund and World Health Organization (2004). Low Birthweight: Country, Regional and Global Estimates.

[29] Suzuki K., Shinohara R., Sato M., Otawa S., Yamagata Z. (2016). Association between Maternal Smoking during Pregnancy and Birth Weight: An Appropriately Adjusted Model From the Japan Environment and Children’s Study. J. Epidemiol..

[30] Veloso H.J., da Silva A.A., Bettiol H., Goldani M.Z., Filho F.L., Simoes V.M., Batista R.F., Barbieri M.A. (2014). Low birth weight in Sao Luis, northeastern Brazil: Trends and associated factors. BMC Pregnancy Childbirth.

[31] Kato T., Yorifuji T., Inoue S., Doi H., Kawachi I. (2013). Association of birth length and risk of hospitalisation among full-term babies in Japan. Paediatr. Perinat. Epidemiol..

[32] Andersen M.R., Simonsen U., Uldbjerg N., Aalkjaer C., Stender S. (2009). Smoking cessation early in pregnancy and birth weight, length, head circumference, and endothelial nitric oxide synthase activity in umbilical and chorionic vessels: An observational study of healthy singleton pregnancies. Circulation.

[33] Roza S.J., Verburg B.O., Jaddoe V.W., Hofman A., Mackenbach J.P., Steegers E.A., Witteman J.C., Verhulst F.C., Tiemeier H. (2007). Effects of maternal smoking in pregnancy on prenatal brain development: The Generation R Study. Eur. J. Neurosci..

[34] Fattal-Valevski A., Toledano-Alhadef H., Leitner Y., Geva R., Eshel R., Harel S. (2009). Growth patterns in children with intrauterine growth retardation and their correlation to neurocognitive development. J. Child Neurol..

[35] Howe L.D., Matijasevich A., Tilling K., Brion M.J., Leary S.D., Smith G.D., Lawlor D.A. (2012). Maternal smoking during pregnancy and offspring trajectories of height and adiposity: Comparing maternal and paternal associations. Int. J. Epidemiol..

[36] Gray T.R., Eiden R.D., Leonard K.E., Connors G., Shisler S., Huestis M.A. (2010). Nicotine and metabolites in meconium as evidence of maternal cigarette smoking during pregnancy and predictors of neonatal growth deficits. Nicotine Tob. Res..

[37] Lin C.C., Su S.J., River L.P. (1991). Comparison of associated high-risk factors and perinatal outcome between symmetric and asymmetric fetal intrauterine growth retardation. Am. J. Obstet. Gynecol..

[38] Garn S.M., Johnston M., Ridella S.A., Petzold A.S. (1981). Effect of maternal cigarette smoking on Apgar scores. Am. J. Dis. Child..

[39] Bosley A.R., Newcombe R.G., Dauncey M.E. (1981). Maternal smoking and Apgar score. Lancet.

[40] Hingson R., Gould J.B., Morelock S., Kayne H., Heeren T., Alpert J.J., Zuckerman B., Day N. (1982). Maternal cigarette smoking, psychoactive substance use, and infant Apgar scores. Am. J. Obstet. Gynecol..

[41] Nijiati K., Satoh K., Otani K., Kimata Y., Ohtaki M. (2008). Regression analysis of maternal smoking effect on birth weight. Hiroshima J. Med. Sci..

[42] Vardavas C.I., Chatzi L., Patelarou E., Plana E., Sarri K., Kafatos A., Koutis A.D., Kogevinas M. (2010). Smoking and smoking cessation during early pregnancy and its effect on adverse pregnancy outcomes and fetal growth. Eur. J. Pediatr..

[43] Secker-Walker R.H., Vacek P.M. (2002). Infant birth weight as a measure of harm reduction during smoking cessation trials in pregnancy. Health Educ. Behav..

[44] England L.J., Kendrick J.S., Wilson H.G., Merritt R.K., Gargiullo P.M., Zahniser S.C. (2001). Effects of smoking reduction during pregnancy on the birth weight of term infants. Am. J. Epidemiol..

[45] Patrick D.L., Cheadle A., Thompson D.C., Diehr P., Koepsell T., Kinne S. (1994). The validity of self-reported smoking: A review and meta-analysis. Am. J. Public Health.

